# RAISE-FER: a massive cross-dataset augmented facial expression dataset

**DOI:** 10.3389/frobt.2026.1757689

**Published:** 2026-03-02

**Authors:** Giuseppe Palestra, Domenico Palmisano, Berardina Nadja De Carolis

**Affiliations:** 1 Hero srl, Martina Franca, Italy; 2 Università degli Studi di Bari “Aldo Moro”, Bari, Italy

**Keywords:** artificial intelligence, AI, computer vision, dataset, facial expression (FE), facial expression recognition (FER)

## 1 Introduction

This data report presents RAISE-FER, a massive cross-dataset augmented facial expression dataset that can be used in social robotics developed during the RAISE (Resilient AI Systems for hEalth) project Palmisano et al. (2025), Palestra et al. (2025) aims at the development of a resilient artificial intelligence system designed to support the elderly population in domestic and care enviroments. The increasing use of robots in everyday or critical contexts has made the need to understand and respond appropriately to human emotions increasingly common. It therefore becomes essential to develop Facial Emotion Recognition (FER) systems capable of being robust and resilient, i.e., able to operate even in adverse or critical conditions such as partial inputs. To support this process, the creation of massive datasets has become necessary with the aim of training the model with compromised or critical data. In the case of FER datasets, it is necessary to create quality and diversified data, also including situations with compromised or corrupted inputs, typical of real-world scenarios. This allows the model, during the operational phase (i.e., in “in-the-wild” scenarios), to better manage these situations and consequently be more resilient and reliable. To support human-robot interaction, Data Augmentation (DA) is necessary and fundamental. In these situations, the purpose of DA is to artificially enrich the dataset in ways that simulate real-world challenges, forcing the model to learn more robust and generalizable features. However, the application of DA in the FER domain requires special attention: the transformations must not alter the perception of the emotion itself. In this work, a complete, structured, and reproducible pipeline for the creation of large-scale augmented datasets is presented, in this case, specifically designed for Facial Emotion Recognition. The entire process has been designed to ensure full reproducibility as described below. The process is carried out using three datasets, but it can be extended and applied to other datasets. The process therefore begins with the union of three existing different datasets: FER2013 Goodfellow et al. (2013), RAF-DB Li et al. (2017), and KDEF Lundqvist et al. (1998). A preprocessing phase is carried out where data unification takes place, standardizing all images to a predefined resolution and filtering the inputs through face detection (MediaPipe Lugaresi et al. (2019)) to discard images without faces. Preprocessing consists of cleaning, standardizing, and unifying the datasets. The cleaning phase is carried out by filtering the dataset using MediaPipe’s face detection. This preliminary operation ensures that every image in our starting set actually contains a detectable face, eliminating corrupt or irrelevant data that could contaminate the training. Subsequently, we applied seventeen distinct transformations to each original image, multiplying the dataset size by 17 times. These transformations include basic techniques implemented with the Albumentations library Buslaev et al. (2020), and advanced techniques such as MixUp, and CutMix and Specific Erasing focused on specific regions like eyes, nose, and mouth, guided by MediaPipe landmarks. During this phase, we explain the two types of transformation:: a first type of transformation where basic transformations are applied, and then where advanced transformations are used. The basic transformations were performed using the Albumentations library and comprise transformation such as rotations, flips, grid distortions, and color alterations—to simulate common variations in pose and camera conditions. The advanced transformations are characterized by MixUp Huang et al. (2025), CutMix Huang et al. (2025), and Specific Erasing. To support these transformations, MediaPipe facial landmarks were used, which are useful for identifying specific facial regions. By applying all distinct transformations to each original image, we exponentially multiply the dataset size.

Finally, another problem is class imbalance derived from basic and advanced transformations. To manage the resulting class imbalance, two further distinct balanced datasets are generated: i) one balanced on the least numerous class; ii) another balanced on the most numerous class by generating new images through basic transformations, randomly varying the transformation parameters. The versions of the dataset created are therefore:

1. RAISE-FER Augmented: This version contains the complete set of images generated after the basic and advanced augmentation phases, without any class balancing applied.
2. RAISE-FER Augmented and Balanced: This version corresponds to the first balancing approach mentioned above, in which the augmented dataset is subsampled to match the least numerous class.
3. RAISE-FER Augmented Balanced Oversampled: This version corresponds to the second balancing strategy, in which the dataset is oversampled to match the most numerous class using random parameter variations.

## 2 Methods

### 2.1 Dataset selection and preprocessing

The first phase focused on the selection of the datasets. The dataset selection was carried out according to criteria of representativeness (presence of the seven basic emotions: happiness, sadness, anger, surprise, disgust, fear, and neutrality), image quality, and availability of consistent annotations.

In this phase, various preprocessing techniques were applied to the three selected datasets (FER2013, RAF-DB, KDEF). Initially, each dataset had a predetermined split into training (train) and testing (test) sets. However, this split was removed to allow a uniform reorganization of the data within the new combined dataset. This choice was motivated by the desire to offer maximum flexibility to the users of the dataset, who can freely choose which portions to include and how to divide it into training, testing, or validation sets according to their own needs.

All three datasets are standardized by resizing all images to 48 × 48 pixels. For RAF-DB, resizing is performed directly since the images are square and resizing to 48 × 48 does not introduce distortions, whereas for the KDEF dataset, a crop is first applied to the height of the image to obtain a square format: the width is taken as a reference, and the upper and lower parts of the image are cropped to obtain a square portion centered on the face. After cropping, the image is resized to 48 × 48 pixels. This operation serves to maintain the face proportions and reduce distortions. Finally, for FER2013, resizing was not performed since the images were already 48 × 48.

In the next step, all images were processed with a face and landmark detection algorithm using MediaPipe, with a confidence threshold set to 0.2. Images in which the face is not detected were discarded. This cleaning operation was necessary because the three datasets contained some images without faces or not depicting people. These images could, in subsequent phases, such as DA, generate additional unnecessary images which could affect the model’s performance.

### 2.2 Data augmentation

After the previous phase, the dataset is augmented through basic and advanced transformations. Reproducibility is ensured for both types of transformations. The choice of techniques to be applied was primarily guided by two objectives: on one hand, to reproduce typical real-world scenarios; on the other hand, to generate new images from those already available while preserving their fundamental characteristics—in this case, maintaining the emotion represented in the image.

#### 2.2.1 Basic transformations

In this phase, the techniques implemented using the Albumentations library are: rotation, horizontal flipping, shearing, grid distortion, random erasing, blurring, random brightness and contrast adjustment, and random color adjustment. These transformations simulate typical real-world situations, such as a tilted face (rotate) or issues with a webcam (random color, blur, etc.). They allow the model to learn to handle common scenarios, becoming more robust and less susceptible to problems. For the transformations rotate, shear, grid distortion, random erasing, random brightness contrast, and random color, the parameters are randomly selected at each application. However, to ensure full reproducibility of the process, metadata related to each transformation are saved in.pkl files. These files contain all the information needed to exactly replicate the same transformations at any time, ensuring that the resulting images are identical to those generated initially. Rotate applies a random rotation to the image between −25° and +25°. Shear performs a combined transformation: scale varies between 80.

#### 2.2.2 Advanced transformations

Advanced transformations include three types: CutMix, MixUp, and Specific Erasing. CutMix combines two images by overlaying a cut-out region from one image onto another. MixUp blends two images together. Specific Erasing consists of erasing precise regions of the image. The regions considered for CutMix and Specific Erasing are the right eye, left eye, nose, and mouth of the subject. To ensure reproducibility of these transformations, the following techniques are applied: for all three transformations, the list of original images to be transformed is ordered according to the counter in the image filename. For MixUp, the images to be blended are taken as ordered pairs starting from the top of the list. The same approach is used for CutMix and Specific Erasing, where, in addition, the coordinates of the regions to be replaced or erased are tracked. MediaPipe is always used to detect the regions of interest, and specific groups of landmarks are identified for each region: i) Left eye: points 300, 293, 336, 285, 463, 261, 265, and 353; ii) Right eye: points 70, 63, 107, 55, 243, 31, 35, and 124; iii) Nose: points 193, 417, 97, and 326 Mouth: points 57, 37, 267, 91, 314, 17, 84, 287, and 321.

These transformations aim, as mentioned, to generate new images from the originals without losing the distinctive characteristics of each image. For example, with CutMix, it is possible to combine parts of the faces of different people (but with the same emotion), generating artificial images that still preserve the same emotional expression.

This approach allows the creation of large-scale datasets starting from a limited initial set, increasing the variety of features in the data. A greater diversity in the dataset enables machine learning models to generalize better, effectively addressing common issues in modern AI related to overfitting or poor generalization.

### 2.3 Application of transformations

For each image in the original dataset, the eight basic transformations and the nine advanced transformations are applied, specifically: one for MixUp, four for CutMix, and four for Specific Erasing, with the latter two applied four times since each transformation targets one of the four considered regions, namely the right eye, left eye, nose, and mouth. This approach allows some transformations considered unnecessary or problematic for the model being trained to be excluded in the future. Applying each of these transformations to the original dataset results in a new dataset 17 times larger.

### 2.4 Balancing

Starting from the new dataset enriched with the 17 transformations per image, an unbalanced dataset is obtained, as some emotions are represented by a much larger number of images than others. To address this issue, two new datasets are created: the first balanced on the class with the fewest images, the second on the class with the most images. In the case of the dataset balanced on the smallest class, the process begins by identifying which emotion has the lowest number of available images. Once identified, all other classes are reduced by selecting only the same number of images, choosing them in an ordered manner based on the numerical counter in each file name and the transformation ID. In this way, each class will have the same number of images and, consequently, the same number of transformations. In the case of the dataset balanced on the largest class, the process begins by identifying the class with the highest number of available images. Then, the classes with fewer images are enriched by adding additional images to them, applying new basic transformations—among those with random parameters—to the original images, namely rotate, shear, grid distortion, random erasing, random brightness contrast, and random color. At this point, both datasets are balanced both in terms of the number of images per class and the type of transformations applied. However, in the dataset balanced on the class with the largest number of images, there is a predominance of basic transformations, since these are the ones that allow for greater variety through controlled parameter randomization. This still ensures good data diversification while maintaining numerical balance between classes.

### 2.5 Data augmentation

**FIGURE 1:**
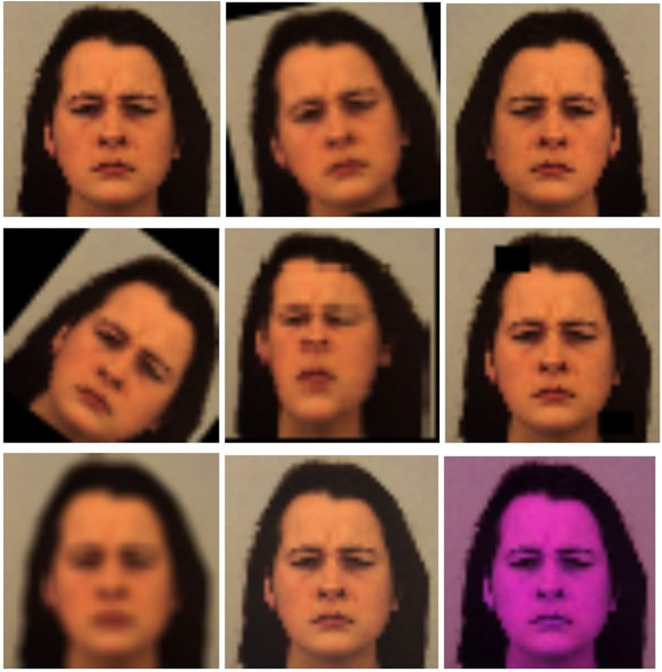
Example of basic transformations.

Below (see Figure 1) are depicted examples of DA techniques applied to some images. The first image is the original one, while the following images each show a transformation applied separately, always starting from the original image. The basic transformations applied, in order, are: rotate, horizontal flip, shear, grid distortion, random erasing, blur, random brightness contrast, and random color.

**FIGURE 2:**
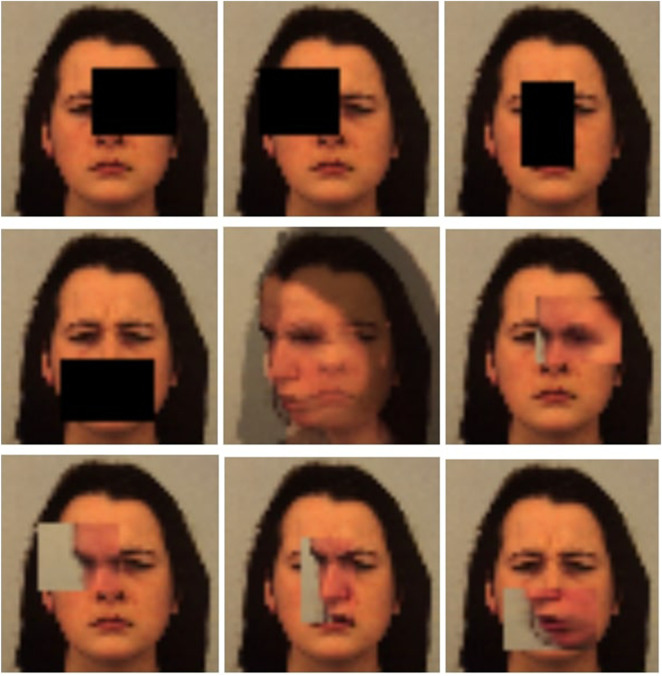
Example of advanced transformations.

In Figure 2 are depicted images part of the advanced transformations, in order (from left to right) are depicted: specific erasing of the left eye, right eye, nose, and mouth. Next, we see the MixUp transformation, and finally the CutMix transformation, both applied to the same four regions of interest under consideration. The post-transformation images retain their validity, as the applied transformations were executed in a controlled manner without completely altering the emotions depicted in the original images. Accordingly, the validated emotional labels can be assumed to remain applicable. Moreover, the adopted data augmentation strategy inherently relies on the inclusion of partially occluded or not fully recognizable images, which are not always individually revalidated, yet are commonly accepted as effective for improving model robustness. Further validation by expert psychologists may nonetheless be beneficial.

## 3 Data analysis

### 3.1 Input datasets characteristics

The datasets used to create the new large-scale dataset for FER namely: FER2013, RAF-DB, KDEF. FER2013 consists of about 35,887 grayscale images, with a resolution of 48 × 48 pixels, and is divided into training and testing sets. The datasets contains the seven basic emotions. RAF-DB consists of about 15,339 RGB images, with a resolution of 100 × 100 pixels, and is divided into training and testing sets. KDEF consists of about 4,900 RGB images, with a resolution of 562 × 762 pixels.

### 3.2 Dataset structure and naming convention

The images that pass the preprocessing filter are saved and renamed using the syntax RAISE_X_Y_C, where X indicates the source dataset (F for FER2013, R for RAF-DB, K for KDEF), Y represents the emotion present in the image (A for angry, D for disgust, F for fear, H for happy, N for neutral, S for sad, SU for surprise), and C is a progressive counter specific to each emotion.

The new dataset is organized into subfolders, each dedicated to a specific emotion, containing the filtered and renamed images. In parallel, a record is kept of all images in which the face was not detected, and a mapping is maintained between the original file names and the new ones, in order to ensure full traceability and replicability of the entire process.

The images generated via augmentation take the base name of the original image, to which the ID of the applied transformation is appended, specifically: one for rotation, two for horizontal flipping, three for shearing, four for grid distortion, five for random erasing, six for blurring, seven for random and contrast adjustment, eight for random coloradjustment, 91 for specific erasing on the left eye, 92 for specific erasing on the right eye, 93 for specific erasing on the nose, 94 for specific erasing on the mouth, 10 for MixUp, 111 for CutMix on the left eye, 112 for CutMix on the right eye, 113 for CutMix on the nose, and 114 for CutMix on the mouth. The new image name is therefore RAISE_X_Y_C_idTransformation.

The generated datasets comprise a total of 3.047 million images distributed in three specific datasets:

- Oversampled Dataset (Balanced to Majority Class): A total of 1.89 million images are included, with 270 thousand images per class.
- Undersampled Dataset (Balanced to Minority Class): A total of 224 thousand images are included, with 32 thousand images per class.
- Augmented Unbalanced Dataset: A total of 933 thousand images. Unlike the balanced sets, this dataset retains the natural class imbalance with the following distribution:
  ○ Angry: 105 thousand images.
  ○ Disgust: 32 thousand images.
  ○ Fear: 101 thousand images.
  ○ Happy: 270 thousand images
  ○ Neutral: 169 thousand images.
  ○ Sad: 151 thousand images.
  ○ Surprise: 105 thousand images.

It was decided not to partition the datasets generated via augmentation into predefined training, testing, or validation sets. This approach aims to provide maximum flexibility to the end user, allowing for the exclusion of specific transformations, identifiable via the naming convention, and enabling custom data splitting strategies to suit diverse experimental requirements.

## 4 Conclusion

In this work, a systematic and integrated approach for the data augmentation of datasets used for Facial Expression Recognition (FER) in HRI was presented. While our specific case utilized three datasets (FER2013, RAF-DB, and KDEF), this approach can be extended to include other datasets. This extension is supported by a consistent preprocessing and standardization phase, which ensures data cleaning and format uniformity.

However, the primary contribution of this research lies not exclusively in the creation of the final unified dataset, but rather in the definition and validation of a highly flexible, modular, and reproducible data processing pipeline. Indeed, the proposed pipeline was designed to be intrinsically extensible; its modular architecture allows future researchers to easily integrate additional source datasets or implement new Data Augmentation techniques both basic and advanced—without the need to restructure the entire workflow. The combined application of geometric and chromatic transformations, alongside semantic mixing techniques such as CutMix and MixUp managed through a metadata tracking system that ensures full reproducibility, offers a concrete solution to the issues of overfitting and poor generalization typical of Deep Learning models trained on limited data. The implementation of this new pipeline, which resulted in the creation of RAISE-FER datasets, stands out significantly from existing datasets such as AffectNet, FER+, and RAF-DB for two main reasons. First, the distinction between widely used FER datasets (FER2013, RAF-DB, KDEF) and RAISE-FER datasets lie in the ability to simulate real-world (in-the-wild) scenarios through transformations, a feature lacking in existing datasets. The adopted approach based on transformations demonstrates that applying data augmentation to several facial expression images can generate robust datasets with variability comparable to massive data collections. This offers the advantage of eliminating the costs and organizational complexity associated with large-scale participant recruitment. The second reason is that we introduce a systematic semantic nomenclature. This allows for the unique identification of each image and provides a clear understanding of exactly which transformations have been applied. This is a major advantage, as it enables future users of the dataset to easily and quickly add or remove image transformations.

## Data Availability

The datasets presented in this study can be found in online repositories. The names of the repository/repositories and accession number(s) can be found below: https://www.kaggle.com/herosrl/datasets.
